# *Piper kelleyi*, a hotspot of ecological interactions and a new species from Ecuador and Peru

**DOI:** 10.3897/phytokeys.34.6376

**Published:** 2014-02-07

**Authors:** Eric. J. Tepe, Genoveva Rodríguez-Castañeda, Andrea E. Glassmire, Lee A. Dyer

**Affiliations:** 1 Department of Biological Sciences, University of Cincinnati, 614 Rieveschl Hall, Cincinnati, Ohio 45221, USA; 2 Department of Integrative Biology, University of Texas at Austin, 1 University Station, Austin, Texas 78712, USA; 3 Biology Department 0314, University of Nevada Reno, 1664 North Virginia Street, Reno, Nevada 89557, USA

**Keywords:** Andes, Braconidae, Ecuador, *Eois*, Geometridae, herbivore-parasitoid interactions, Piperaceae, *Piper* section *Macrostachys*, plant-herbivore interactions, Tachinidae

## Abstract

We describe *Piper kelleyi* **sp. nov.**, a new species from the eastern Andes of Ecuador and Peru, named in honor of Dr. Walter Almond Kelley. *Piper kelleyi* is a member of the *Macrostachys* clade of the genus *Piper* and supports a rich community of generalist and specialist herbivores, their predators and parasitoids, as well as commensalistic earwigs, and mutualistic ants. This new species was recognized as part of an ecological study of phytochemically mediated relationships between plants, herbivores, predators, and parasitoids. Compared to over 100 other *Piper* species surveyed, *Piper kelleyi* supports the largest community of specialist herbivores and parasitoids observed to date.

## Abstract (es)

Describimos la nueva especie *Piper kelleyi* **sp. nov**., proveniente de la vertiente Este de los Andes en el Ecuador y Perú, y nombrada en honor al Dr. Walter Almond Kelley. *Piper kelleyi* forma parte del clado *Macrostachys* del género *Piper* y conforma la base alimenticia de una diversa comunidad de herbívoros, tanto generalistas como especialistas, depredadores y parasitoides de estos herbívoros, así como tijeretas comensales y hormigas mutualistas. Esta nueva especie fue reconocida como parte de una investigación ecológica de las interacciones, mediadas por fitoquímica, entre plantas, herbívoros, depredadores y parasitoides. En comparación con más de otras 100 especies de *Piper* estudiadas, *Piper kelleyi* hospeda la comunidad de insectos con mayor diversidad de herbívoros especialistas y parasitoides observada hasta ahora.

## Introduction

Documenting plant and phytochemical diversity is recognized as an important component of understanding interactions among plants, herbivores and predators, and for understanding the evolution of biodiversity (Ehrlich and Raven 1964). Ongoing ecological, evolutionary, and phytochemical studies that focus on the plant genus *Piper*, and its insect herbivores and mutualists, have revealed a network of complex interactions in which plant phytochemistry has an evolutionary impact across multiple trophic levels (Dyer and Palmer 2004, Miller and Dyer 2009, Wilson et al. 2012, Dyer et al. 2013). Through the course of these studies, dozens of new species of the *Piper* specialist lepidopteran herbivore *Eois* Hübner (Geometridae: Larentiinae) and their parasitoids have been discovered (Grinter et al. 2009, Inclán-Luna 2010, Strutzenberger et al. 2010, Inclán-Luna and Stireman 2013). Among the *Piper* species studied thus far, the one that supports the highest diversity of *Eois* and associated parasitoids is also a previously unrecognized species. In this paper we describe this new species of *Piper* from Ecuador and Peru, and discuss the diversity of organisms that it sustains.

## Materials and methods

### Morphology and species distribution modeling

Morphological data and locality information were taken from the personal collections of EJT, GRC, and AEG, the ecological studies mentioned above, and from specimens sent to EJT for identification. Although the new species occurs in Ecuador and Peru, the distribution model was restricted to Ecuador since the majority of collection data is from the northeast and southeast Andean slopes of Ecuador. Collection density was very high in Napo Province, Ecuador (where much of the project activity was focused) relative to other parts of the species range, and to avoid biased results due to this imbalance, locations were filtered so that the model was based on an even sample from across the species’ range. The distribution model was calculated using the maximum entropy method (Maxent software package; Phillips et al. 2006) from 26 presence locations marked with GPS and 19 climatic parameters at the 25km^2^ scale, which were downloaded from WorldClim (www.worldclim.org; Hijmans et al. 2005). The model was calibrated using elevations at which the species is modeled to be present, against elevations at which the species was observed in the field. The methods are described fully in Rodríguez-Castañeda et al. (2012).

### Natural history studies

Study sites were located in the northeastern Andes of Ecuador in the provinces of Napo and Sucumbíos along an elevational transect from Amazonian lowland rain forest (200 m) to Andean páramo (3200 m). Mean temperature along this gradient drops linearly with elevation at a rate of approximately -0.5°C per 200 m elevation gain, and precipitation ranges from 3900–4500 mm yr^-1^ (Rodríguez-Castañeda et al. 2010). This area includes three mountain ranges: the Sumaco, Huacamayos and Reventador, as well as continuous lowland to montane rain forests (sensu Holdridge 1967) that are part of the Cayambe–Coca, Antisana, and Gran Sumaco nature reserves.

To document trophic interactions associated with the new *Piper* species, standard 10 m diameter plots were established inside the forest at the sampling sites (Dyer et al. 2010a). A mean of 9 ± 0.81 plots were sampled at 200 m intervals along an elevational range from 280–3200 m; the new species was encountered from 1200–2300 m along this transect. Inside each plot, the leaves and stems of all individuals of *Piper* present were harvested (leaving the roots intact) kept in a separate bag identified by plot number and plant number, and transported to laboratories at Jatun Sacha (400 m), Yanayacu Biological Station (2200 m), or Oyacachi (3200 m) for further study. At the laboratory, all leaves were carefully surveyed for caterpillars or other insects. When the plants had ants or other arthropods inside the petioles, these were collected and placed in 75% ethanol in labeled vials. Each caterpillar found chewing on *Piper* leaves was assigned a unique number, identified to morphospecies, and reared to an adult moth for identification, or in the case of parasitized caterpillars, to obtain parasitism rates and identify the parasitoids.

## Taxonomic treatment

### Piper kelleyi

Tepe sp. nov.

urn:lsid:ipni.org:names:77135933-1

http://species-id.net/wiki/Piper_kelleyi

Figs 1 –2

#### Diagnosis.

Piperi imperiali *(Miq.) C.DC. similis sed sinu foliorum laxe aperto (numquam angusto neque petiolum tegente) et tuberculis leviter elevatis differt*.

#### Type.

**Ecuador:** Prov. Napo, Cantón Quijos, island in the Cosanga River near Las Palmas, 0°32'42"S, 77°52'36"W, 1875 m, 19 Jan 2009 (fl), E.J. Tepe & W. Simbaña 2615 (holotype: QCNE; isotypes: MO, MU).

#### Description.

Shrub to small tree, 1.5–15 m tall, moderately branched; trunk of flowering individuals 5–8 cm d.b.h.; some individuals with prop roots. Stems glabrous, the nodes moderately to densely tuberculate, density of tubercules increasing distally along each internode, the internodes 6.5–9.6 cm long and 0.5–1.1 cm in diam.; shoot apex emerging from the sheathing leaf base. Prophyll minute and hidden by the sheathing leaf base. Leaves more or less distichous on flowering branches, with petioles 6–14 × 0.7–1 cm at flowering nodes, vaginate, and with persistent margins, the margins extending to the leaf base or projecting 1–10 mm beyond the insertion of lower leaf lobe, glabrous, sparsely to moderately tuberculate; laminae 25–50 × 15–42 cm, broadly ovate, the apex obtuse, rounded, the base oblique, cordate, the lobes equal or more commonly somewhat unequal, extending 2–8 cm below the petiole attachment, divergent and never overlapping the petiole, the sinus open and the apices of the lobes nearer the leaf margin than the petiole, the sides of the lamina arising 7–8 mm apart on the petiole, the lamina drying thickly chartaceous, densely glandular-dotted (usually blackish on dried specimens, 3–5 per mm^2^, increasing in density along the leaf margin), glabrous above, glabrous to sparsely pubescent on the lamina below and moderately to densely pubescent on the veins below with much shorter trichomes (< 0.2 mm long; these more conspicuous on the secondary and tertiary veins, often lacking on the midvein), the 4–6 pairs of major secondary veins arising from the lower 2/3 of the midvein, arcuate-ascending, primary–tertiary veins somewhat impressed above, prominent below. Inflorescences free from the leaf base of the same node, pendulous, 40–72 × 0.4–0.8 cm in flower and 0.8–1.1 cm in diameter in fruit, the flowers densely crowded and +/- banded; peduncles 2.5–6 × 0.25–0.5 cm, white to green in fruit, glabrous; floral bracts 0.7–1.0 mm broad, triangular to triangular-rounded, nearly glabrous with upper margin white ciliate to densely pubescent throughout; stamens 4 per flower, the stamens maturing asynchronously such that only one or two are apparently visible per flower at one time, white, the anthers 1–1.5 mm long, the filaments clavate, the connective somewhat broadened between thecae and these divergent at ca. 45°, dehiscing laterally; fruits rounded or rectangular from above by compression, 1.5–2 × 1.2–2 mm, the apex truncate, glabrous, gland-dotted, stigma lobes 3(–4), 0.5–0.8 mm long, sessile or on a very short style, caducous; seeds rounded-square, flattened, 1.5–2 mm.

**Figure 1.**
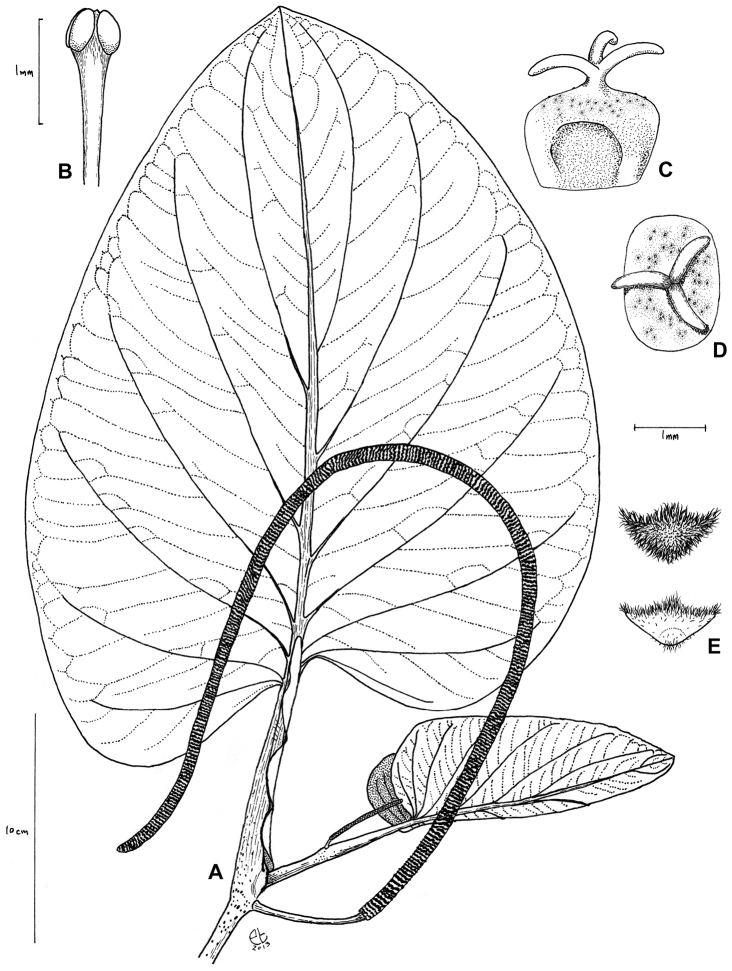
*Piper kelleyi* Tepe. **A** leaf and inflorescence **B** Stamen **C** fruit in lateral view **D** Fruit in apical view **E** Bracts in apical view. [**A** and **E** (lower) drawn from Tepe et al. 1597; **B–E** (upper) drawn from Tepe et al. 2615]

**Figure 2.**
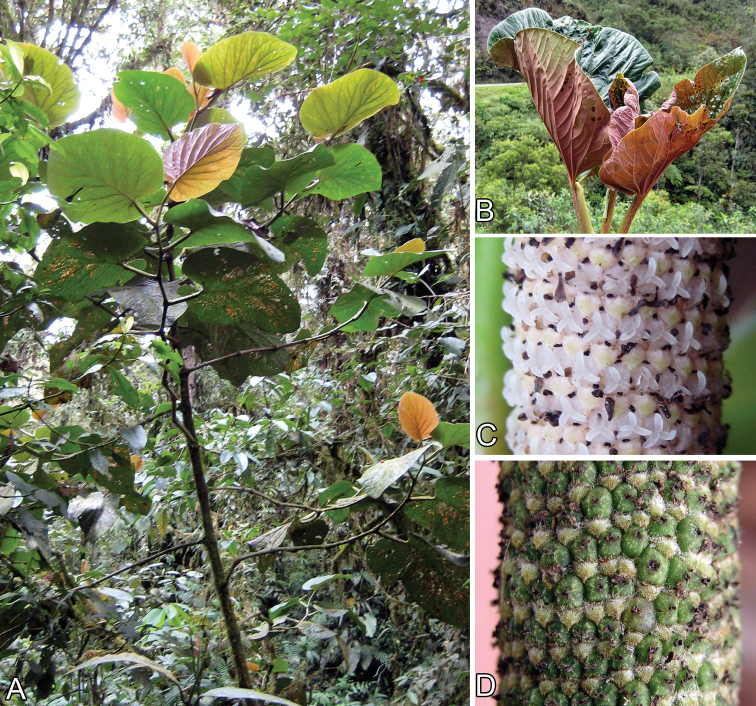
*Piper kelleyi*. **A** Habit **B** Close-up of leaves showing characteristic red color of younger leaves [Tepe et al. 2381] **C** Close-up of inflorescence [Tepe et al. 2615] **D** Close-up of infructescence [Tepe et al. 1597].

#### Distribution.

**Figure 3.**
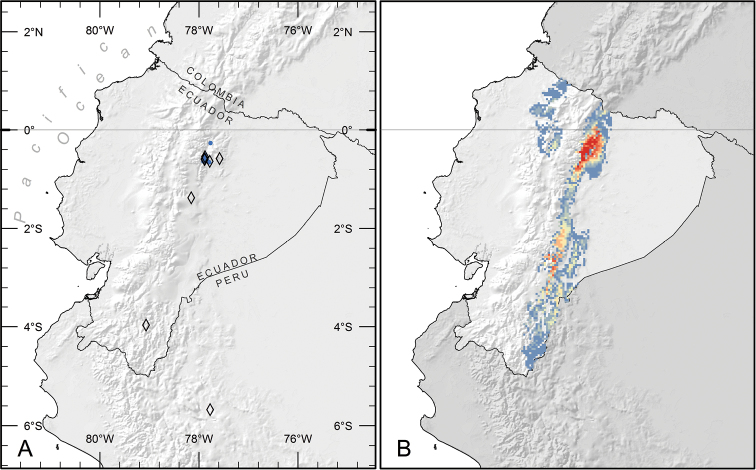
Distribution of *Piper kelleyi*. **A** Distribution of known localities based on collections (open diamonds) and study plots used to gather natural history data (blue circles) **B** Predicted distribution based on analysis of habitat parameters using the maximum entropy method. Areas in red are the most likely to have suitable habitat for *Piper kelleyi* (probability = 0.75–0.87), and areas in blue (0.02–0.08) or unlabeled (< 0.02) are the least likely.

*Piper kelleyi* is found in shaded understory habitats of primary and secondary lower montane rainforests (Neill 1999) on the eastern slopes of the Andes in Ecuador and northern Peru; elevation 1200–2400 m (Fig. 3A).

#### Phenology.

Flowering specimens have been collected in Jan–Mar and Dec; fruiting specimens have been collected in Mar, Jul, and Sep.

#### Etymology.

*Piper kelleyi* is named for Dr. Walter Almond Kelley (1942–2010). Dr. Kelley spent a good portion of his career focusing on taxonomy within the genus *Cryptantha* (Boraginaceae; e.g., Kelley and Wilken 1993), but became fascinated with *Piper* when he visited Costa Rica in 1997 to work with *Piper* species at La Selva Biological Station. He was interested generally in angiosperm evolution, evolution of unique morphologies, stem anatomy, and tropical biology and spent years working with *Piper* from Costa Rica and Ecuador (e.g., Tepe et al. 2009). He examined the unique morphology of *Piper*, and documented *Piper* stem anatomy. He had made considerable progress on understanding phyllotaxy and the notoriously complicated stem anatomy in *Piper*, and produced two substantive manuscripts on *Piper* biology. His extensive observations on *Piper* morphology, included the following comments:

There are three basic patterns of sympodial stem tip and leaf primordial (STLP) enclosure and protection in *Piper*. The first common pattern has the sympodial STLP enclosed in a prophyll only. A second common pattern has the sympodial STLP enclosed in a prophyll and a stipular wrap-over of the terminal subtending leaf. A third, rarer pattern occurs when the prophyll has become greatly reduced so that the sympodial STLP appears to be enclosed only by a stipular wrap-over of the terminal subtending leaf (W.A. Kelley, field journal).

*Piper kelleyi* exhibits the third of these patterns. He established the herbarium (MESA) at Colorado Mesa University, Grand Junction, Colorado (CMU), and cultivated a high diversity of *Piper* species at the CMU greenhouse, including *Piper kelleyi*. These plants are still used for research today.

#### Preliminary conservation status.

According to the IUCN Red List Categories (IUCN 2013), we deem it necessary to classify *Piper kelleyi* as Data Deficient (DD). *Piper kelleyi* appears to occupy a relatively narrow elevational band along the eastern slopes of the Andes in Ecuador and northern Peru, but is fairly common in Ecuador between 1400–2200 m in Napo and between 1900–2000 m in Zamora-Chinchipe Provinces. These two areas have established field stations with projects focusing on *Piper* and, accordingly, collection of *Piper* has been comparatively intense in these areas. Scattered collections of *Piper kelleyi* in other parts of its range, however, indicate the species’ presence, but are not necessarily informative of its abundance. Collecting intensity across the Neotropics is decidedly uneven (Schulman et al. 2007) and virtually nothing is known about the density of individuals of this species outside of the two focus areas mentioned above. Consequently, we are presently unable to make an informed statement regarding the conservation status of *Piper kelleyi*.

#### Specimens examined.

**ECUADOR. Napo:** Yanayacu Biological Station, 3 km SW of Cosanga, 0°36'S, 77°53'W, 2080 m, 24 Sep 2005 (fr), J. Homeier, C. Chicaiza & B. Moreno 1646 (GOET, QCA, QCNE); Parque Nacional Sumaco-Galeras, southern slope of Sumaco Volcano, 0°35'S, 77°35'W, 1930 m, 19 Mar 2008 (fr), J. Homeier, M.A. Chinchero, E. Jaramillo & D. Simba 3362 (GOET, QCA, QCNE); Parque Nacional Sumaco-Galeras, Río Hollín watershed, 0°38.3'S, 77°46.9'W, 1490 m, 28 Mar 2008 (st), J. Homeier, M.A. Chinchero, D. Simba, L. Guachamin & M. Unger 3524 (GOET, QCA, QCNE); Cosanga, on road to the Yanayacu Biological Station, 0°35'09"S, 77°53'04"W, 2100–2200 m, 2 Mar 2006, (fl, fr), E.J. Tepe et al. 1597 (MO, MU, QCNE); Canton Quijos, ca. 4 km W of Cosanga on the Cosanga - Las Caucheras road, 0°35'52.1"S, 77°53'10.0"W, 2120 m, 16 Feb 2011 (fl), E.J. Tepe & M.P. Moreno 2999 (MO, QCA, QCNE); Parque Nacional Sumaco-Galeras, southern slope of Sumaco Volcano, 0°35'S, 77°35'W, 2015 m, 21 Nov 2006 (st), S. Trogisch, S. Moritz & J. Homeier 312 (GOET, QCA, QCNE). **Tungurahua:** Zuñag Scientific Station, 1°22'41"S, 78°09'20"W, 1581 m, 19 Jul 2012 (fr), A. Glassmire, M. Habdas & A. Crespin B13 (CINC). **Zamora-Chinchipe**: Reserva San Francisco, 3°58'S, 79°04'W, 1950 m, 6 Sep 2008 (st), N. Cumbicus & J. Peña *643* (GOET, LOJA); Reserva San Francisco, road Loja-Zamora, ca. 35 km from Loja, 3°58'S, 79°04'W, 2000 m, 20 Mar 2009 (st), M. Ebinghaus 6 (MU). **PERU. Amazonas:** Road from Rioja to Pedro Ruiz, km 383 (old white markers), 5°40'39"S, 77°46'24"W, 2000 m, 16 Dec 2007 (infl), E.J. Tepe, S. Leiva, S. Stern & M. Zapata Cruz 2381 (MU, USM). The specimen database is available at http://hdl.handle.net/2374.UC/731268 .

## Results and discussion

*Piper kelleyi* is a striking species that stands out from its surroundings because of its long, white, pendulous inflorescences, large leaves, and the pinkish coloration characteristic of the young stems and leaves. These colorful young leaves have given this species the whimsical, informal name “pink belly,” which was used in two previous works (O’Connor 2011, Wilson et al. 2012).

*Piper kelleyi* is a member of the *Macrostachys* clade of *Piper*, species of which are most abundant in the shady understory of lowland and cloud forest habitats in the Andes and Central America (Jaramillo et al. 2008). Species of this clade have a prophyll that is highly reduced and completely obscured by the sheathing petiole, but can be most easily recognized by the typically large leaves with leaf bases that are strongly asymmetric and often prominently lobed, and the long, pendulous inflorescences that characterize most species. *Piper kelleyi* can be distinguished from other members of the clade by the unusually broad sinus between the basal lobes of the leaf (Fig. 1). In *Piper kelleyi* the lobes are always divergent and never overlap the petiole, whereas in most species of this clade, including *Piper imperiale* (Miq.) C.DC., which is the species perhaps most likely to be confused with *Piper kelleyi*, the sinus is often narrow and one of the basal leaf lobes sometimes covers the petiole. Aside from leaf characters, this new species can be distinguished by the slightly raised tubercles on the stems and petioles (or their complete absence), as compared to the often conspicuously raised tubercles of *Piper imperiale* (as much as 5 mm or more in some extreme specimens). The other species of *Piper* sect. *Macrostachys* that is likely to co-occur with *Piper kelleyi* is *Piper marsupiiferum* Trel.; however, this species is a slender shrub, short in stature (tall plants can reach 1.5 m with the majority being less than 1 m tall), with narrow, strongly asymmetric, deeply rugose leaves with a basal lobe that completely covers the petiole. In addition, the inflorescences of *Piper marsupiiferum*, while pendulous, are a maximum of 15 cm long (vs. 40–72 cm in *Piper kelleyi*). Other large-leaved pipers that co-occur with *Piper kelleyi*, at least in the northern part of its range, include *Piper baezanum* C.DC., which has coarsely rugose, symmetrical leaves, and the lianescent *Piper schuppii* A.H. Gentry. Furthermore, both of these species are distinct from *Piper kelleyi* in that they have inflorescences that are held upright during all stages of development.

### Distribution

Ecological niche model performance was high (avg. train AUC: 0.99; avg. test AUC: 0.99; max. probability range: 0–0.86). Results predict that suitable habitat for *Piper kelleyi* is more extensive than the distribution reported here, which is based on available collections and observations (Fig. 3B). The species favors montane elevations (ca. 1000–2500 m) and according to the model, important determinants of its habitat were intolerance to freezing temperatures and affinity for temperate climate (coldest month temperatures of ~12.5°C). Further habitat suitability increases with high levels of precipitation during the coolest months (>1000 mm). The species seems to favor river or stream banks and sandy soils (pers. observation by GRC). The model predicts suitable habitat on both sides of the Andes, but *Piper kelleyi* has never been recorded from localities west of the continental divide despite intensive *Piper* collection by EJT and surveys of *Piper* for caterpillars by AEG at several localities on the western slopes of the Andes. These collections included seven species of *Piper* sect. *Macrostachys*, but did not include *Piper kelleyi*. It is possible that additional collecting will reveal the presence of the new species in these areas, but based on current collections it appears that *Piper kelleyi* is restricted to limited localities on the eastern slopes of the Andes.

### Ecology

*Piper kelleyi* is a focal component of a study aimed at understanding the influence of plant secondary chemistry on herbivores and their associated parasitoids (Wilson et al. 2012). The genus *Piper* produces a high diversity of multiple classes of secondary compounds, including biologically active amides and imides (Dyer et al. 2004), which are known to negatively affect generalist herbivores (Dyer et al. 2003). Preliminary evidence suggests that *Piper kelleyi* is not palatable to generalist herbivores based on assays conducted by GRC and AEG during studies spanning 2005–2008 and 2011–2013 respectively (unpubl. data).

**Figure 4.**
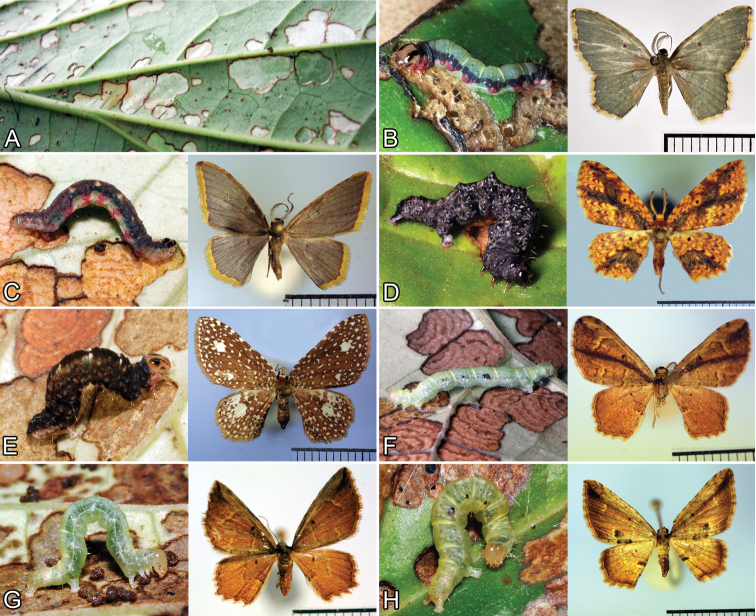
**A** Leaf of *Piper kelleyi* with characteristic herbivory marks or “windows” made by the specialist herbivore *Eois* species of *Eois* that specialize on *Piper kelleyi* include **B** *Eois viridiflava* (Dognin) **C** *Eois* aff. *viridiflava* (Dognin) **D** *Eois ignefumata* (Dognin) **E** *Eois planetaria* (Dognin) **F** *Eois* aff. *pallidicosta* (Warren) **G** *Eois encina* (Dognin) **H** *Eois* aff. *encina* (Dognin).

Plot data resulted in herbivory and parasitism rates, and measures of herbivore and parasitoid richness. Mean herbivory rates for the plant are close to 20%, and the only herbivores that are regularly found on *Piper kelleyi* are species of the specialist herbivore *Eois*. Even in the absence of caterpillars, the presence of *Eois* can be identified by the characteristic feeding markings that they leave behind (see the herbivory key in Dyer et al. 2010b). These caterpillars scrape small portions of the under sides of leaves, leaving unpigmented windows (Fig. 4A) that are eventually lost, resulting in characteristic holes in the leaves. Apart from *Eois*, other lepidopteran herbivores reared from this host plant include members of t the families Erebidae, Hesperiidae, Noctuidae, Pyralidae, and Tortricidae. Over 3,000 caterpillars have been reared from *Piper kelleyi*, yet only single individuals have been recorded from these other lepidopteran families (Dyer et al. 2013) suggesting that, unlike *Eois*, they are not likely to be *Piper* specialists.

Project collaborators have reared nearly 30,000 caterpillars and parasitoids from over 100 *Piper* species, and *Piper kelleyi* is the host species with highest density and species richness of *Eois* found thus far. Since many of the *Eois* species and nearly all of the parasitoids reared from *Piper* were previously undescribed, the taxonomy of these insect groups currently lags behind that of their host plants. Nevertheless, *Piper kelleyi* hosts at least 11 morphospecies of the specialist herbivore *Eois* and these include *Eois cancellata* (Warren), *Eois encina* (Dognin), *Eois* aff. *encina* (Dognin), *Eois goodmani* (Schaus), *Eois ignefumata* (Dognin), *Eois* aff. *necula* (Druce), *Eois olivacea* (Felder & Rogenhofer), *Eois* aff. *pallidicosta* (Warren), *Eois planetaria* (Dognin), *Eois viridiflava* (Dognin), and *Eois* aff. *viridiflava* (Dognin) (Fig. 4B–H; Rodríguez-Castañeda 2009, Dyer et al. 2013: www.caterpillars.org ). Seven of these species were recognizable from adult characters as distinct species, whereas additional cryptic species were recognized from a combination of molecular data and larval characteristics (Wilson et al. 2012). These results are not surprising since molecular identification has increased Geometrid estimates of diversity by 50% in the SE Andes of Ecuador (Strutzenberger et al. 2010).

The parasitism rate for all of the *Eois* collected from *Piper kelleyi* was 8.63%. Parasitic wasps were the most frequent and included Eulophidae (Hymenoptera: Chalcidoidea) at 3.5%, followed by Braconidae (Microgastrine) at 2.7%, and Ichneumonidae at 1.5%. Tachinid flies (Diptera: Tachinidae) had the lowest parasitism rate at 0.9%. Within the subfamily Microgastrine (Braconidae), species of the genera *Cotesia* (Cameron),* Glyptapanteles* (Ashmead), *Mesochorus* (Gravenhorst), *Parapanteles* (Ashmead), and *Protopanteles* (Ashmead) have been reared out of the *Eois* species feeding on *Piper kelleyi*. The determined Tachinid species include *Erythromelana abdominalis* (Townsend), *Eois cryptica* (Inclán & Stireman), and *Eois jaena* (Townsend) (Inclán-Luna 2010, Dyer et al. 2013, Inclán-Luna and Stireman 2013).

**Figure 5.**
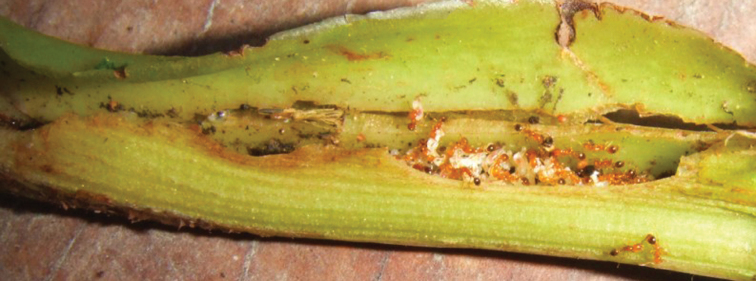
A colony of *Pheidole* ants nesting inside a petiole of *Piper kelleyi*.

Thus, to date, *Piper kelleyi* is host to at least 17 species of specialist and generalist herbivores, and nine described and an estimated 20–30 undescribed species of parasitoids (unpubl. data). Moreover, *Piper kelleyi* acts as a microcosm for other insect species. The sheathing leaf petioles occasionally host nests of *Pheidole* (Westwood) (Hymenoptera: Formicidae; Fig. 5), other ants, or small families of earwigs. Several species of the *Macrostachys* clade of *Piper* have specialized associations with ants that nest primarily within the petioles (Risch et al. 1977, Letourneau 1998, Tepe et al. 2004); however, when the large sheathing petioles of other species form a sufficiently closed chamber, a diversity of opportunistic, arboricolous ants take advantage of these chambers for nesting sites (Tepe et al. 2007). Also, predatory coccinellid beetle larvae and salticid spiders are frequently found on the leaves of *Piper kelleyi* (GRC, personal observation).

### Conclusions

Empirical evidence has been accumulating that supports the hypothesis that plant biochemistry helps drive the evolution of biodiversity, not only among herbivores that feed directly on plants, but among higher trophic levels as well (Ehrlich and Raven 1964, Forister and Feldman 2011, Wilson et al. 2012). *Piper kelleyi* represents a hotspot of these interactions. In fact, our group has surveyed lepidoptera feeding on *Piper* species for over 20 years, and *Piper kelleyi* supports the highest diversity of *Eois* recorded to date. We have documented a large number of parasitoids as well, but, because parasitism rates are low and discoveries are still occurring, we expect the number of parasites to be similarly high.

Our description of *Piper kelleyi* is part of a larger effort to combine traditional taxonomy and natural history with newer integrative approaches. The purpose of this project is to successfully contribute to taxonomy and systematics while characterizing evolutionary patterns via molecular phylogenetics, studying community ecology, and increasing our understanding of functional diversity by quantifying specialized consumer-resource relationships and interaction diversity. The importance of traditional taxonomy and natural history data for these studies is essential (as argued in Dyer et al. 2010a) and descriptions of new species, such as *Piper kelleyi*, are a necessary component of thorough research programs in ecology and evolutionary biology.

## Supplementary Material

XML Treatment for Piper kelleyi
